# Additional Evidence for *DDB2* T338M as a Genetic Risk Factor for Ocular Squamous Cell Carcinoma in Horses

**DOI:** 10.1155/2019/3610965

**Published:** 2019-09-15

**Authors:** Moriel H. Singer-Berk, Kelly E. Knickelbein, Zachary T. Lounsberry, Margo Crausaz, Savanna Vig, Nikhil Joshi, Monica Britton, Matthew L. Settles, Christopher M. Reilly, Ellison Bentley, Catherine Nunnery, Ann Dwyer, Mary E. Lassaline, Rebecca R. Bellone

**Affiliations:** ^1^Veterinary Genetics Laboratory, School of Veterinary Medicine, University of California-Davis, Davis, CA 95616, USA; ^2^Veterinary Medical Teaching Hospital, University of California-Davis, Davis, CA 95616, USA; ^3^Bioinformatics Core Facility, Genome Center, University of California-Davis, Davis, CA 95616, USA; ^4^Insight Veterinary Specialty Pathology, Davis, CA, USA; ^5^Department of Surgical Services, School of Veterinary Medicine, University of Wisconsin-Madison, Madison, WI 53706, USA; ^6^School of Veterinary Medicine, University of Pennsylvania, Philadelphia, PA 19104, USA; ^7^Genesee Valley Equine Clinic, Scottsville, NY 14546, USA; ^8^Department of Surgical and Radiological Sciences, School of Veterinary Medicine, University of California-Davis, Davis, CA 95616, USA; ^9^Department of Population Health and Reproduction, School of Veterinary Medicine, University of California-Davis, Davis, CA 95616, USA

## Abstract

Squamous cell carcinoma (SCC) is the most common periocular cancer in horses and the second most common tumor of the horse overall. A missense mutation in *damage-specific DNA-binding protein 2* (*DDB2*, c.1012 *C*>*T*, p.Thr338Met) was previously found to be strongly associated with ocular SCC in Haflinger and Belgian horses, explaining 76% of cases across both breeds. To determine if this same variant in *DDB2* contributes to risk for ocular SCC in the Arabian, Appaloosa, and Percheron breeds and to determine if the variant contributes to risk for oral or urogenital SCC, histologically confirmed SCC cases were genotyped for the *DDB2* variant and associations were investigated. Horses with urogenital SCC that were heterozygous for the *DDB2* risk allele were identified in the Appaloosa breed, but a significant association between the *DDB2* variant and SCC occurring at any location in this breed was not detected. The risk allele was not identified in Arabians, and no Percherons were homozygous for the risk allele. High-throughput sequencing data from six Haflingers were analyzed to ascertain if any other variant from the previously associated 483 kb locus on ECA12 was more concordant with the SCC phenotype than the *DDB2* variant. Sixty polymorphisms were prioritized for evaluation, and no other variant from this locus explained the genetic risk better than the *DDB2* allele (*P* = 3.39 × 10^−17^, *n* = 118). These data provide further support of the *DDB2* variant contributing to risk for ocular SCC, specifically in the Haflinger and Belgian breeds.

## 1. Introduction

Ocular squamous cell carcinoma (SCC) is the second most common tumor to affect the equine eye and in severe cases can lead to loss of vision or loss of life. Previous research investigating the genetic risk for ocular SCC in the Haflinger breed identified a recessive mode of inheritance and a 483 kb locus on equine chromosome 12 (ECA12: 11284011-11767256) that was associated with limbal and nictitating membrane SCC in the Haflinger breed [1–3]. Sanger sequencing of a candidate gene from this locus identified a risk variant in *damage-specific DNA-binding protein 2* (*DDB2*, c.1012 *C*>*T* p.Thr338Met, rs1139682898). This variant was strongly associated, but not perfectly concordant, with limbal and third eyelid SCC status in both the Haflinger and Belgian breeds, explaining 76% of the Haflinger and Belgian cases investigated [2–4]. A recent case report also implicated this *DDB2* variant as a potential risk factor for ocular SCC in Rocky Mountain Horses [5]. The first aim of this study was to gain further insight into ocular SCC development in Haflinger and Belgian breeds by investigating other risk factors, including age.

The etiopathogenesis for ocular SCC is not fully understood, but hormones and pigmentation have been suggested to contribute to risk for this disease. Retrospective studies have shown that there is a sex bias among horses diagnosed with SCC [6], with a greater distribution of males diagnosed with ocular SCC than females [7–11]. When the population of affected horses was distributed by sex and castration status, geldings were more commonly affected followed by mares and then stallions [7–10]. It was hypothesized that castrated males are more likely to develop SCC because of a lack of circulating androgens and estrogens that are present in mares and stallions [12, 13]. Although this hypothesis has yet to be fully investigated, it could provide additional insight into the apparent predisposition for SCC in geldings. In a recent study investigating risk factors in Belgian horses, there were significantly more affected males than unaffected males (*P*_males_ = 0.039). However, because only two stallions were available for that study (one case and one control), investigating if geldings were more often affected compared to stallions was not possible [4]. In this study, we also aim to investigate sex and castration status as a risk variable for ocular SCC phenotype in Haflingers, the breed for which we have the largest well phenotyped data set.

Several retrospective studies have evaluated coat color phenotypes for a potential connection to ocular SCC. In a group of studies on ocular SCC, the most frequently reported base coat color among the affected samples was chestnut [9–11, 14]. Depending on breed, between 32% and 46% of the affected SCC population was identified to have a chestnut base coat color, suggesting that coat color pigmentation, and specifically a variant in *MC1R* (which is known to cause the chestnut phenotype), may predispose horses to ocular SCC development.

Aside from the available data for Haflinger and Belgian breeds, the genetic mechanisms underlying ocular SCC in horses has not been investigated, despite reports describing high occurrences in several breeds including the Appaloosa, Arabian, American Paint Horse, American Quarter Horse, and various draft breeds [7–9]. This suggests that genetics may also play a role in these additional breeds. A previous study investigated the frequency of the *DDB2* variant (*T*) in several breeds of horses that were reported to have a high incidence of ocular SCC, but the horses in that study were not phenotyped for SCC status. That study also identified the presence of the risk allele, albeit at a low frequency, in the Appaloosa (0.02) and the Percheron (0.07, [2]), suggesting that the *DDB2* variant may also contribute to the risk of ocular SCC in these additional breeds. While the *DDB2* risk allele was not identified in the Arabian horse population screened, the Haflinger breed is thought to have been derived from a half Arabian stallion, suggesting this mutation arose after the foundation of the Haflinger breed. Contrary to this, the presence of this variant in multiple diverse breeds suggests that *DDB2* c.1012 *C*>*T* arose prior to the development of the Haflinger breed and that some Arabians may therefore be at an elevated risk for ocular SCC due to this variant [15]. Therefore, we also aimed to investigate if *DDB2* c.1013 *C*>*T* is a genetic risk factor for ocular SCC in the Appaloosa, Percheron, and Arabian breeds in horses that had been clinically confirmed to have the disease.

In addition to ocular locations, SCC also has been reported to occur around or within the oral cavity and the urogenital regions in the horse [16, 17]. SCC is commonly reported to occur on the penis and prepuce in males and the perianal, vulvar, and clitoral regions in females [7, 16, 18]. Breeds including the Appaloosa, Arabian, and American Paint Horse are frequently diagnosed with urogenital SCC, suggesting a genetic component may be at play. SCC of the oral cavity is rarely diagnosed but, when reported, is typically localized to the lips and tongue [19, 20]. While the precise etiology of oral and urogenital SCC is unknown, equine papillomavirus type 2 (EcPV2) and lack of photoprotective pigmentation have been implicated as risk factors [21–23]. This is a distinction between ocular SCC and urogenital SCC as only one periocular SCC case has been reported to be positive for papillomavirus; this case was positive for both bovine PV and EcPV2. While several studies have reported the presence of EcPV2 detected in urogenital cancers, in one study, as high as 43% of penile SCC cases were identified to be positive for EcPV2 [24, 25]. Given the breed-specific reports, we propose a genetic mechanism may also play a role in SCC development at these mucosal membrane locations.

The previously identified associated risk variant for ocular SCC in Haflingers and Belgians is a missense mutation in *DDB2* (c.1013 *C*>*T* p.Thr338Met), which codes for a protein involved in repair of UV-damaged DNA. UV irradiation is a well-known risk factor for SCC development in horses and other species [12] because of the propensity to induce DNA damage in the form of 6-4 photoproducts (6-4PP) and cyclobutane pyrimidine dimers (CPD) [26]. If these lesions occur in protein-coding genes involved in cell cycle regulation or DNA replication or repair, dysregulated cell growth and tumor development may occur [27]. The protein encoded by *DDB2* recognizes and binds to these damaged DNA lesions, recruiting other proteins to repair them [28, 29]. The missense mutation in equine *DDB2* (c.1013 *C*>*T* p.Thr338Met) was computationally predicted to be deleterious and is hypothesized to alter the shape of the DNA damage recognition and binding domains, affecting this protein's ability to bind and repair damaged DNA. While the predicted function and the strong association with limbal and third eyelid SCC support this as the causal variant, this variant was not perfectly concordant with the ocular SCC disease phenotype. Specifically, one unaffected Haflinger individual, last examined at age twenty, was homozygous for the risk allele, and to date, 10 Haflingers and 6 Belgians affected with ocular SCC are either homozygous for the reference allele or heterozygous for the associated *DDB2* risk variant [2–4]. Thus, it is possible that the *DDB2* allele is not the causal risk allele and instead is tagging another variant from the 483 kb associated haplotype on ECA12. The final aim of the study utilized a high-throughput resequencing approach to determine if another variant within the previously associated haplotype on ECA12 was perfectly concordant with the ocular SCC phenotype in the Haflinger breed.

## 2. Methods

### 2.1. Animals and Phenotyping

Blood and hair samples were collected from horses with owner's written consent, and formalin-fixed paraffin-embedded (FFPE) tissue samples were obtained from those banked at the University of California-Davis Veterinary Medical Teaching Hospital's Anatomic Pathology Service. Affected samples were collected from horses with SCC at ocular (limbal, third eyelid, and eyelid), urogenital (prepuce, sheath, vulva, penis, perirectal, scrotum, and clitoris) and oral regions (mandible, pharynx, maxilla, and tongue). Inclusion criteria for affected samples were histopathologic confirmation of a diagnosis of SCC. For ocular controls, horses were confirmed to be unaffected for ocular SCC via ophthalmic examination by a Diplomate of the American College of Veterinary Ophthalmologists and were at least 13 years of age at the time of examination, which was one standard deviation above the mean age of diagnosis as reported previously for limbal SCC in the Haflinger breed [1]. A table describing samples (those presented previously and new samples) by breed, phenotype, and SCC location can be found in [Supplementary-material supplementary-material-1].

### 2.2. Whole Genome Sequencing Sample Selection

Six Haflinger horses (four cases and two controls) were selected for whole genome sequencing based on ocular SCC status, age, *DDB2* c.1013 *C*>*T* T338M genotype, and ECA12 haplotype analysis (Table 1) [2]. To identify potential variants perfectly concordant with phenotype, the individuals selected for sequencing represented the cases and controls that could provide the most information in downstream analysis. Specifically, two cases homozygous for the *DDB2* variant (*T/T*) that defined the break points of the 483 kb run of homozygosity on the 5′ and 3′ end, respectively, were utilized [2]. The two other affected horses, selected for sequencing, were genotyped as homozygous reference (*C/C)* for the *DDB2* variant, and one of them had a haplotype that was comprised mostly of case minor alleles, while the other had mostly case major alleles (Table 1). All four cases selected were confirmed to have SCC of the limbus or limbus and nictitans. One of the controls sequenced had the expected homozygous reference genotype (*C/C)* for the *DDB2* risk variant, while the other was the only unaffected Haflinger (out of 118 tested to date) that was homozygous for the associated *DDB2* risk allele *(T/T)*.

### 2.3. DNA Extraction and *DDB2* Genotyping

Genomic DNA was extracted from blood or hair follicles using the Puregene whole-blood extraction kit (QIAGEN Inc., Valencia, CA) following the manufacturer's and previously reported protocols [2]. Genomic DNA was extracted from FFPE tissue using either the GeneRead DNA FFPE kit or the Quick-DNA FFPE kit (Zymo Research) following the manufacturer's protocols. Genotyping the *DDB2* variant was performed using the commercially available assay at the University of California Davis Veterinary Genetics Laboratory. As FFPE DNA extractions are known to result in DNA of low integrity, genotypes were only recorded for those samples in which genotyping was also possible for at least one microsatellite marker with a larger polymerase chain reaction (PCR) product size and one microsatellite with a smaller PCR product size than that of the *DDB2* PCR assay.

### 2.4. Sequencing, Bioinformatics, and Variant Prioritization

Library preparation and whole genome high-throughput sequencing were carried out at the UC Berkeley QB3 Vincent J Coates Genomics Sequencing Facility using the Illumina HiSeq 4000 platform (Illumina, San Diego, CA, USA) generating 150 base-pair paired-end reads. Preprocessing of the sequence reads was done using expHTS (version 1.0.1) [30], which included filtering duplicates, trimming reads based on quality, concatenating overlapping R1 and R2 reads, and removing sequencing adapters. The processed reads were aligned to EquCab2, the most up-to-date assembly when the analysis was performed, using the Burrows-Wheeler Aligner (BWA mem version 0.7.16, [31]), and SAMtools (version 1.6) was used for post alignment processing. Variant calling was carried out using FreeBayes (version 1.1) [32] and SAMtools mpileup [33] using default parameters. Variants were annotated with the Ensembl annotation (GCA_000002305.1), and the predicted effects were determined using SnpEff (version 4.3i) [34].

**Figure 1:**
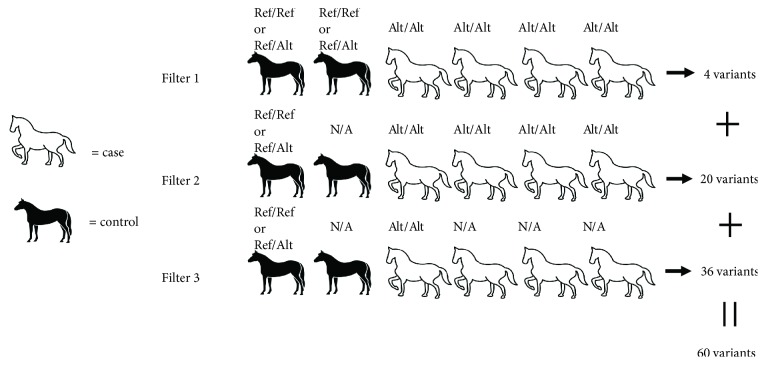
Filtering of variants from high-throughput Illumina sequencing analysis of six Haflingers phenotyped for ocular SCC. These filtering parameters were established for the most robust analysis to identify any potential variant from the associated ECA12 haplotype that may explain the prevalence for ocular SCC in Haflinger horses. Filter 1 was used to categorize variants based on the strictest parameters in order to identify variants more concordant with the ocular SCC phenotype than the *DDB2* variant. Filter 2 used slightly less strict filtering of variants by allowing one of the control horses to genotype homozygous alternate for that variant. Lastly, filter 3 used the least stringent filtering by allowing for one or more affected horses to genotype as homozygous reference or heterozygous for that variant. On the diagram, N/A signifies any genotype.

Variants were filtered for the previously associated region of homozygosity plus one megabase in either direction (ECA12:10284011-12767256). Variants were prioritized for further evaluation based on three different filtering criteria (Figure 1). Filter 1 specifically addressed our final aim, that is, using data from all six horses sequenced to identify variants perfectly concordant with the phenotype from the previously associated haplotype region. All variants identified with this filter were prioritized for further evaluation. Because it is possible that an individual may have the genetic risk for cancer but may not develop tumors, filter 2 was less stringent with respect to our control genotypes, allowing one control to be homozygous alternate for any given variant. Any nonsynonymous variant that was predicted to influence function (high, moderate, or low) was prioritized for further analysis. Variants annotated as modifiers from filter 2 were also prioritized for further genotyping if they were called by both variant callers (SAMtools and FreeBayes), and they were located within the 483 kb associated region of interest (ECA12: 11284011-11767256), narrowing the total of prioritized modified variants from this filter from 299 to 17. Finally, filter 3 was performed in an effort to identify all possibly functional variants and allow for less than perfect concordance with the phenotype in cases compared to controls. For this filter, only one case had to be homozygous alternate and at least one control had to be have a reference allele. Only those nonsynonymous variants called by both callers were prioritized. The calls of the prioritized variants from these three filtering strategies were visually confirmed from alignments using the Integrative Genomics Viewer (v.2.3.91), and variants which appeared to also map to an alternate location in the genome or that were located in highly repetitive regions (e.g., instances of more than two alleles present and/or extremely deep sequencing relative to surrounding sequence) were removed [35, 36].

After the completion of this analysis, a new assembly of the horse genome was released (EquCab3) [37]. Because the aim of this project was to identify a variant perfectly concordant with the phenotype, we remapped the whole genome sequencing data to the new assembly and investigated variants from the filter 1 parameters described above.

### 2.5. Genotyping of Prioritized Variants from Whole Genome Sequencing

A total of sixty variants were selected based on the filtering criteria described above ([Supplementary-material supplementary-material-1] and Table 2). Variants genotyped, genomic locations, alleles, corresponding genes, and primer sequences can be found in Tables [Supplementary-material supplementary-material-1], [Supplementary-material supplementary-material-1], [Supplementary-material supplementary-material-1], and [Supplementary-material supplementary-material-1]. Fifty-seven of these variants were successfully designed for Agena MassARRAY Spectrophotometry (Agena Bioscience Inc., San Diego, CA, USA) and genotyped at the Veterinary Genetics Laboratory at the University of California, Davis. However, one variant genotyped with MassARRAY had a call rate of zero, and to verify high-throughput sequencing results, this variant (12:g.12442525A>G) was Sanger sequenced in all six samples utilized to generate Illumina sequencing reads ([Supplementary-material supplementary-material-1]). Amplicons were purified using EdgeBio Quickstep 2 PCR purification kit following the manufacturer's protocol (EdgeBio, Gaithersburg, MD, USA). The purified amplicons were sequenced using BigDye Terminator v3.1 and ABI 3730 Genetic Analyzer. The sequencing data were analyzed with Sequencher version 5.2.4 (http://www.genecodes.com).

The three variants that could not be designed for MassARRAY genotyping were genotyped by other PCR genotyping methods. Variant 12:g.12004291C>T was genotyped by a PCR restriction fragment length polymorphism assay (PCR-RFLP) using the enzyme BbvI (New England, Biolab Inc., Ipswich, MA, USA). A 12 base-pair insertion (12:g.10594033_10594034insAGCAGCTCCAGC) was genotyped by analyzing the PCR product using an ABI 3730 Genetic Analyzer (Applied Biosystems, at Thermo Fisher Scientific, Grand Island, NY, USA), which is capable of resolving this allele size difference. PCR reactions for 12:g.10594033_10594034insAGCAGCTCCAGC and 12:g.12004291C>T were performed using a standard PCR protocol using 0.1 U of FastStart Taq DNA polymerase (Roche Applied Science, Indianapolis, IN, USA) and FAM-labelled primers, which enabled detection of PCR products. The remaining variant (12:g.12318425_12318441insT) was validated by allele-specific PCR in the six horses that were used for the whole genome high-throughput sequencing analysis ([Supplementary-material supplementary-material-1]).

### 2.6. Statistical Analysis

The *DDB2* risk variant was previously found to be associated with ocular SCC in both the Haflinger and Belgian breeds [2–4]; thus, we combined data from these two breeds to have additional power to evaluate age as a risk factor across breeds. An unpaired *t*-test was used to determine if there was a significant difference between the mean ages of Haflingers and Belgians diagnosed with ocular SCC with respect to their genotype classes. A chi-squared test of association was also performed to determine if sex was a risk factor for ocular SCC in Haflingers (*n* = 120) for whom we had sex information. As there were only two stallions (1 case and 1 control) in the Belgian data set, we evaluated castration status as a risk factor only in the Haflinger data set. Fisher's exact test was performed to determine if there was a significant difference in the number of geldings and stallions between cases and controls for the 49 male Haflingers with available data. Fisher's exact test was also performed to compare the allele frequencies for the *DDB2* variant in the SCC Appaloosa sample set with a random sampling of unphenotyped Appaloosa horses from Bellone et al. [2].

Association testing of variants prioritized from the resequencing analysis was performed using the Golden Helix SNP and Variation Suite (version 8.3.0, Golden Helix Inc., Bozeman, MT, USA; http://www.goldenhelix.com). For SNP association testing, SNPs with a call rate < 90% (*N* = 5) or minor allele frequency < 0.05 were excluded (*N* = 7). Chi-squared tests of association under a recessive model were performed. The four variants most concordant with ocular SCC phenotypes were analyzed further using the Ensembl genome browser, raw genotype data, and PredictSNP to determine and analyze the effect of each polymorphism [38].

## 3. Results

### 3.1. Investigating Risk Variables for Ocular SCC

A total of 120 Haflingers and 43 Belgians, including those horses used in previous studies ([Supplementary-material supplementary-material-1]) [2, 3], were used to determine if sex and age were contributors to risk for ocular SCC. Haflinger and Belgian horses affected with ocular SCC and homozygous for the *DDB2* c.1013 *C*>*T* variant were significantly younger than those affected Belgian and Haflinger horses with one or no copies of the alternate allele (mean age = 10.13 verses 13.14, *P* = 0.04, Table 3). There was no statistical difference in the number of males and females regarding SCC status in the population of Haflingers (*P* = 0.73, [Supplementary-material supplementary-material-1]). However, we did detect significantly more affected geldings than stallions compared to the control sample set (*P* = 0.02, Table 4). Two Percherons (one male and one female) diagnosed with ocular SCC were carriers for the *DDB2* risk variant, and no homozygotes were detected. Only three affected Percherons were identified for this study, and thus, an association could not be tested ([Supplementary-material supplementary-material-1]). The *DDB2* risk allele was not detected in any of the 46 Appaloosas or 19 Arabians that were phenotyped for ocular SCC and included in this study ([Supplementary-material supplementary-material-1]).

### 3.2. Investigating DDB2 c.1012 *C*>*T* as a Risk Factor for Oral and Urogenital SCC

Eight oral SCC cases were identified from the Arabian (*n* = 2), Appaloosa (*n* = 4), and Haflinger (*n* = 2) breeds, and none were found to possess the *DDB2* risk variant ([Supplementary-material supplementary-material-1]). A total of seventy-three urogenital SCC cases were evaluated from Arabians (*n* = 13) and Appaloosas (*n* = 60, [Supplementary-material supplementary-material-1]), none of which were homozygous for the *DDB2* risk variant. These data do not support a role for the *DDB2* variant in urogenital cancer risk in these breeds. Four out of sixty Appaloosas with urogenital SCC were heterozygous for the *DDB2* variant; however, the allele frequency did not significantly differ in this population compared to that of the random unphenotyped population reported in Bellone et al. (*P* = 1.0, Table 5) [2].

### 3.3. Investigating Variants on ECA12 for Association with Ocular SCC in Haflingers

The final aim of this work was to determine if a single variant from the ECA12 SCC associated locus was perfectly concordant with the ocular SCC phenotype in Haflinger horses. Using our strictest filtering parameter and mapping to EquCab2, five variants were identified that were perfectly concordant with the phenotype in the six horses sequenced (Table 2 and [Supplementary-material supplementary-material-1]). 12:g.12517018_12517026insGTTTGTTT was excluded following visual inspection using the Integrative Genomics Viewer, as there were many reads containing the alternate allele with a mapping quality score of zero. The four remaining variants were further scrutinized ([Supplementary-material supplementary-material-1]). None were predicted to influence protein function, three were annotated as intron variants, and one (12:g.12442525A>G) was found in an intergenic region and was the only variant using this filtering parameter that was called by both variant callers. Sanger sequencing of 12:g.12442525A>G and allele-specific-PCR of 12:g.12318425_12318441insT could not replicate genotype calls identified by the high-throughput sequencing analysis, and these two variants were not further considered. The two remaining filter 1 variants were genotyped in a large cohort of phenotyped samples. SNP 12:g.11760227T>G was not as concordant with ocular SCC phenotype as the *DDB2* variant (*P* = 0.04). SNP 12:g.11956103A>G was the only filter 1 variant from the EquCab2 analysis that was also identified when data were remapped to EquCab3 (Supplemental [Supplementary-material supplementary-material-1]). This variant was removed from the final data analysis due to low minor allele frequency detected in the large cohort genotyped by the MassARRAY assay. One additional variant was identified by mapping to EquCab3 that met our filter 1 criteria (EquCab3 12:g.11935134T>C). This variant was predicted by SnpEff to be a modifier and is an intron of *CUGBP Elav-like family member 1* (CELF1), a gene suspected to be involved in myotonic dystrophy type 1 [39]. This variant was not investigated further.

Three-hundred and ninety-one variants were identified by our filter 2 analysis, and twenty of them were prioritized for further investigation (Table 2). Two of these were predicted to have a moderate effect on protein function, and one was predicted to have a low effect. One of the variants predicted to have a moderate effect and the variant predicted to have a low effect on protein function were called by both variant callers (12:g.10544757T>C and 12:g.12064892A>G), while the other moderate variant (12:g.12004293C>T) was unique to SAMtools. 12:g.12064892A>G was predicted to be an intronic splice region variant, while 12:g.10544757T>C, LARGE2 p.Leu347Pro, and 12:g.12004293C>T, FNBP4 p.Ala815Thr, were both predicted to be missense variants. Most of the variants from this filtering parameter (98%) were predicted by SnpEff to be modifiers (*N* = 384). To prioritize these potential modifying variants for association testing with ocular SCC phenotype, variants called by both variant callers and those that were found in the strict region of interest (ECA12:11284011-11767256) were scrutinized further. A total of seventeen modifiers were selected for further interrogation ([Supplementary-material supplementary-material-1]). Of the seventeen modifiers, eleven were intronic variants and six were intergenic variants.

Over 5000 variants were identified by the filter 3 analysis (Table 2). Twenty-five variants, not identified previously in filter 2, were called by both variant callers and were predicted to have a moderate effect on their corresponding protein function. As expected, based on our filtering parameters and previous work on *DDB2*, one variant identified by this analysis was the *DDB2* c.1013 *C*>*T* variant. This *DDB2* variant along with the other 24 variants predicted to have a moderate effect on protein function were prioritized for further analysis. Additionally, we also prioritized low effect variants from this filter and synonymous variants (*n* = 55) were excluded for follow-up investigation, leaving a total of eleven predicted low effect variants ([Supplementary-material supplementary-material-1]).

### 3.4. MassARRAY Genotyping and Association Testing

**Figure 2:**
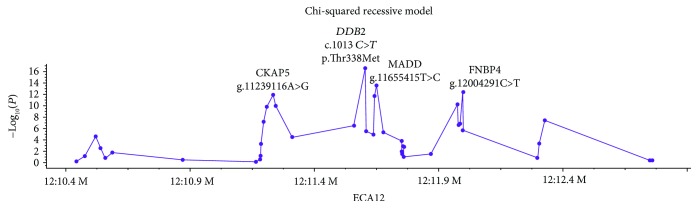
Association testing of variants identified from analysis of resequencing data in Haflingers phenotyped for ocular SCC. The *DDB2* variant (c.1013 *C*>*T*) was the most concordant with ocular SCC phenotype in a population of 118 Haflingers (56 cases and 62 controls). Other variants that were strongly associated but less concordant with ocular SCC phenotype include 12:g.11239116A>G (CKAP5), 12:g.11655415T>C (MADD), 12:g.12004291C>T (FNBP4). -log_10_*P* values for each polymorphism are presented for the sample set.

Of the fifty-seven variants on the MassARRAY, five were removed from association testing based on low call rates and an additional seven were filtered for low minor allele frequency. One other variant was removed after filtering because of insufficient cardinality, resulting in a total of 47 variants that passed quality control filtering. Using a chi-squared test of association under a recessive model, the *DDB2* SNP (c.1013 *C*>*T*) was the most concordant with ocular SCC phenotype (*P* = 3.39 × 10^−17^, Figure 2). Three other SNPs that were identified by filter 3 (12:g.11655415T>C, 12:g.12004291C>T, and 12:g.11239116A>G) were highly concordant with the ocular SCC phenotype, although less so than the *DDB2* variant (*P*_12:g.11655415T>C_ = 3.95 × 10^−14^, *P*_12:g.12004291C>T_ = 4.81 × 10^−13^, and *P*_12:g.11239116A>G_ = 1.68 × 10^−12^). Analysis of these three variants in conjunction with the *DDB2* genotype did not support multiple variants from this locus contributing to risk, as all three of these SNPs had more controls with the risk associated genotype than the *DDB2* variant (Table 6). Further, only 12:g.12004291C>T, FNBP4 p.Ala815Cys, was classified as a missense mutation by SnpEff. However, this variant was predicted to have a neutral consequence by the consensus classifier PredictSNP, with a confidence score of 83%, thus rendering it unlikely to be causal.

## 4. Discussion

Our results do not support the *DDB2* variant (c.1012 *C*>*T*, p.Thr338Met, rs1139682898) as a major genetic risk factor for ocular SCC in the Appaloosa, Arabian, or Percheron breeds. However, given the lower frequency of the *DDB2* variant in these breeds, it is still plausible that this variant explains a small percentage of the risk for ocular SCC, and testing additional individuals is necessary to definitively exclude this variant as a risk factor in these breeds. Our results do not support the *DDB2* variant as a genetic risk factor for urogenital SCC in the Appaloosa and Arabian breeds, as no homozygotes were detected in the sixty Appaloosa horses and thirteen Arabian horses tested. These data suggest that, at least for the Appaloosa and Arabian breeds, urogenital SCC is not associated with the ocular SCC risk variant and other genetic variants or etiologic factors may be at play. For example, EcPV2 infection has been shown to explain a large percentage of urogenital cases [21, 24]. Similarly, none of the eight oral SCC cases were homozygous for the *DDB2* risk allele; however, our available sample set was small.

The mean age of diagnosis was significantly younger for ocular SCC-affected Haflinger and Belgian horses who were homozygous for the *DDB2* variant compared to those that were heterozygous or homozygous for the reference allele (10 years of age versus 13). Sex was not significantly associated with SCC status in the Haflinger data set; however, there were significantly more geldings than stallions affected with ocular SCC. This is consistent with previous reports in the literature, which suggest that geldings may be predisposed to SCC [7–10]. These data propose that hormones could be an important risk variable in ocular SCC development in Haflinger horses, though further analysis is required to fully understand this association [12, 13].

Whole genome sequencing of six Haflingers identified sixty prioritized variants for genotyping in a larger cohort to determine if another variant within the 483 kb region on ECA12 might be more perfectly concordant with the ocular SCC phenotype than the previously identified *DDB2* variant. Of these sixty prioritized variants, none were more perfectly concordant with the disease phenotype than the *DDB2* variant. Of the four variants identified by filter 1 (perfectly concordant in the 6 horses sequenced), quality control checks (genotype verification and filtering for MAF) eliminated three of these variants from further investigation. The one remaining filter 1 variant was not as strongly associated with the ocular SCC phenotype as the *DDB2* variant when testing our full data set. All four of these filter 1 variants plus the one additional variant identified in the EquCab3 analysis ([Supplementary-material supplementary-material-1] and [Supplementary-material supplementary-material-1]) were predicted to be modifiers, and a sound hypothesis of their role in cancer development could not be generated based on their known gene function in other organisms. In contrast, the *DDB2* variant is predicted to have a deleterious effect on protein function. Taken together, the variants identified in filter 1 are unlikely to be contributing to predisposition for ocular SCC in the Haflinger breed.

While not as concordant with the SCC phenotype as the *DDB2* variant, three other variants identified in filter 3 (12:g.11655415T>C, 12:g.12004291C>T, and 12:g.11239116A>G) were highly concordant with the ocular SCC phenotype (*P* < 3.95 × 10^−14^) and were predicted to have moderate effect on protein function. 12:g.12004291C>T, FNBP4 p.Ala815Cys, was identified by SnpEff as a missense variant with a moderate effect; however, PredictSNP software identified this variant as neutral with 83% accuracy. This protein plays a role in the regulation of cytoskeletal dynamics during cell division and migration as well as the maintenance of membrane curvature at sites of new vesicle formation [40]. While not previously implicated in cancer development, mutations in this gene in other species, namely, humans and mice, have been associated with Waardenburg anophthalmia syndrome, a disease characterized by anophthalmia and microphthalmia, and limb abnormalities [41]. Haflinger horses have not been reported nor observed by our research team to have ocular anomalies consistent with Waardenburg syndrome, and therefore, this variant is unlikely contributing to cancer risk.

12:g.11655415T>C and 12:g.11239116A>G were predicted to be splice variants and are both located in introns of genes implicated in tumor development in humans. 12:g.11655415T>C was identified by SnpEff as a splice region variant in *MAP kinase activating death domain* (*MADD*). MADD is involved with propagation of apoptotic signals and regulates cell proliferation [42]. 12:g.11239116A>G was also identified as a splice region variant in a gene that has been previously implicated in colon and hepatic cancers in humans. Specifically, this variant is found in intron 31 of 43 of the *cytoskeleton-associated protein 5* (*CKAP5*). CKAP5 plays a role in spindle formation, specifically protecting the kinetochore microtubules from depolymerization, as well as centrosomal microtubule assembly [43]. The expression of both MADD and CKAP5 is not known in equine SCC nor has alternative splicing of these genes been investigated in the horse. To definitively rule out these variants as contributors to cancer risk, transcriptional studies are warranted. Although the full effects of these splice region variants are unknown, if they contribute to ocular SCC risk in the Haflinger, it is not to the same degree as the *DDB2* variant.

## 5. Conclusion

Given the known role that UV damage plays in cancer development, a mutation in the protein *damage-specific DNA-binding protein 2* is likely detrimental to repair UV-damaged DNA. The nature of the *DDB2* variant and its strong association with the ocular SCC phenotype, the predicted deleterious effect of this variant on protein function, and the evidence presented here that no other variant was identified that better explains cancer risk provide strong evidence that *DDB2* c.1013 *C*>*T* p.Thr338Met is the causal genetic risk factor for ocular SCC in the Haflinger and Belgian breeds. Additional functional assays will help to further test our hypothesis that this missense variant impairs DDB2 from recognizing and binding to UV-damaged DNA. These data also provide support for the specific utilization of genetic testing to screen horses for this variant to assist with clinical management and breeding decisions. As this *DDB2* variant is not perfectly concordant with the ocular SCC phenotype, genetic heterogeneity could explain additional cases of Haflinger and Belgian horses.

## Figures and Tables

**Table 1:** Haflinger samples selected for whole genome sequencing based on phenotype, age, *DDB2* c.1013 *C*>*T* genotype, and ECA12 haplotype analysis.

Sample	Status	*DDB2* genotype	Age at ocular exam	Features for selection
HF-14-62	Affected limbal SCC	*C/C*	13	Haplotype with majority case minor alleles
HF-14-55	Affected limbal SCC	*C/C*	6	Haplotype with majority case major alleles
HF-13-25	Affected limbal SCC	*T/T*	9	Defined 3′ breakpoint of haplotype
HF-15-07	Affected limbal and nictitating membrane SCC	*T/T*	12	Defined 5′ breakpoint of haplotype
HF-13-17	Unaffected	*C/C*	27	Oldest sample at the time of submission
HF-13-33	Unaffected	*T/T*	20	Risk haplotype

**Table 2:** Number of variants identified and prioritized for further evaluation based on three filtering parameters and mapping to EquCab2.

Variants from filtering		Unique to FreeBayes	Unique to SAMtools	Identified by both variant callers	Prioritized variants
Filter 1	High	0	0	0	0
	Moderate	0	0	0	0
	Low	0	0	0	0
	Modifier	3	1	1	4
Filter 2	High	0	0	0	0
	Moderate	0	1	1	2
	Low	0	0	5 (4—synonymous)	1
	Modifier	38	47	299	17
Filter 3	High	0	0	0	0
	Moderate	27	28	25	25
	Low	2	3	66 (55—synonymous)	11
	Modifier	316	415	4759	0

**Table 3:** Comparison of mean age of ocular SCC diagnosis in Haflingers and Belgians (*n* = 83) with respect to *DDB2* c.1013 *C*>*T* genotypes.

	*C/C* or *C/T*	*T/T*
Mean age	13.14	10.13
Standard deviation	6.39	5.37
Standard error of the mean	1.36	0.69
Total	22	61
*P* value = 0.04		

**Table 4:** Investigating castration status and risk for ocular SCC in a population of Haflingers.

	Gelding	Stallion	Total
Ocular SCC affected	24	1	25
Unaffected	17	7	24
Total	40	8	49
*P* value = 0.02			

**Table 5:** *DDB2* c.1013 *C*>*T* genotypes in SCC phenotyped populations versus an unphenotyped sample set of Appaloosa horses.

SCC locations	*C/C*	*C/T*
Ocular SCC	46	0
Oral SCC	4	0
Urogenital SCC	56	4
Total	106	4
Reference population (unphenotyped for SCC)^∗^	96	3

^∗^Bellone et al., 2017.

**Table 6:** Association between ocular SCC affection status and CKAP5 12:g.11239116A>G, MADD 12:g.11655415T>C, and FNBP4 12:g.12004291C>T genotypes.

*CKAP5* 12:g.11239116A>G	*A/A* or *A/G*	*G/G*	Total
Affected	7	49	56
Unaffected	48	14	62
Total	55	63	118
*P* value = 1.68 × 10^−12^			
*MADD* 12:g.11655415T>C	*T/T* or *C/T*	*C/C*	Total
Affected	12	44	56
Unaffected	56	6	62
Total	68	50	118
*P* value = 3.95 × 10^−14^			
*FNBP4* 12:g.12004291C>T, p.Ala815Cys	*C/C* or *C/T*	*T/T*	Total
Affected	10	45	55
Unaffected	52	9	61
Total	62	54	116
*P* value = 4.81 × 10^−13^			

## Data Availability

The whole genome sequencing data used to support the findings of this study have been deposited in the European Nucleotide Archive repository (PRJEB30871).

## Supplementary Materials

Supplementary Materials Table S1: samples and affection status for of horses utilized in this study. Table S2: primer sequences and PCR conditions for variants confirmed by Sanger sequencing or genotyped by PCR-RFLP analysis. Table S3: variants prioritized for evaluation from filter 1 and their EquCab2 coordinates, polymorphism, Reference SNP ID, gene, variant type, and caller utilized to identify the variant. Table S4: variants identified from remapping to EquCab3 and using filter 1 parameters. SNP ID, Reference SNP ID, EquCab3 position, polymorphism, gene, variant type, and caller utilized to identify variant are described. Table S5: variants prioritized for further evaluation using filter 2 criteria (mapped to EquCab2) and their relative position, alleles, gene, and predicted functional effect. Table S6: prioritized variants from filter 3, genomic coordinates, alleles, gene, and predicted effect. Figure S1: flow diagram of variants from prioritization through analysis. Table S7: investigating gender with respect to SCC affection status in Haflingers. Table S8: ocular SCC cases by breed and their *DDB2* (c.1013 *C*>*T*) genotypic distribution. Table S9: breeds diagnosed with oral SCC and their respective genotypes for the *DDB2* variant c.1013 *C*>*T*. Table S10: urogenital SCC sample information and *DDB2* c.1013 *C*>*T* genotypes.
