# Supplementary material for: Additional Evidence for DDB2 T338M as a Genetic Risk Factor for Ocular Squamous Cell Carcinoma in Horses
Source: Int J Genomics. 2019 Sep 15;2019:3610965. doi: 10.1155/2019/3610965 (PMC6766160; doi:10.1155/2019/3610965)
Supplement: Supplementary Materials — Table S1: samples and affection status for of horses utilized in this study. Table S2: primer sequences and PCR conditions for variants confirmed by Sanger sequencing or genotyped by PCR-RFLP analysis. Table S3: variants prioritized for evaluation from filter 1 and their EquCab2 coordinates, polymorphism, Reference SNP ID, gene, variant type, and caller utilized to identify the variant. Table S4: variants identified from remapping to EquCab3 and using filter 1 parameters. SNP ID, Reference SNP ID, EquCab3 position, polymorphism, gene, variant type, and caller utilized to identify variant are described. Table S5: variants prioritized for further evaluation using filter 2 criteria (mapped to EquCab2) and their relative position, alleles, gene, and predicted functional effect. Table S6: prioritized variants from filter 3, genomic coordinates, alleles, gene, and predicted effect. Figure S1: flow diagram of variants from prioritization through analysis. Table S7: investigating gender with respect to SCC affection status in Haflingers. Table S8: ocular SCC cases by breed and their DDB2 (c.1013 C>T) genotypic distribution. Table S9: breeds diagnosed with oral SCC and their respective genotypes for the DDB2 variant c.1013 C>T. Table S10: urogenital SCC sample information and DDB2 c.1013 C>T genotypes. [file 3610965.f1.pdf]

International Journal of Genomics

Additional Evidence for *DDB2* T338M as a Genetic Risk Factor for Ocular Squamous Cell Carcinoma in Horses

Moriel H. Singer-Berk[1], Kelly E. Knickelbein[1,2], Zachary T. Lounsberry[1], Margo Crausaz[1], Savanna Vig[1], Nikhil Joshi[3], Monica Britton[3], Matthew L. Settles[3], Christopher M. Reilly[4], Ellison Bentley[5], Catherine Nunnery[6], Ann Dwyer[7], Mary E. Lassaline[8], Rebecca R. Bellone[1,9]

Supplemental Material

Table S1: Samples and affection status for horses utilized in this study.

Samples used Previously*	Breed	Number Affected (SCC all locations)				Number Unaffected
		Ocular	Urogenital	Oral	Other	
96	Haflinger	47	0	0	0	49
43	Belgian	25	0	0	0	18
**New Samples**						
27	Haflinger	12	2	0	0	13
109	Appaloosa	45	59	3	2**	0
33	Arabian	18	12	2	1***	0
3	Percheron	3	0	0	0	1

*Bellone *et al*., 2017, Singer-Berk *et al*., 2018, and Knickelbein *et al*., 2019
**Included in this analysis were two Appaloosas with multiple SCC diagnosis. One was affected at both the ocular and urogenital regions and the other at the ocular and oral regions.
***There was also one Arabian included that was confirmed affected with SCC of both ocular and urogenital regions.

Table S2: Primer sequences and PCR conditions for variants confirmed by Sanger sequencing or genotyped by PCR-RFLP analysis.

SNP ID	Forward Primer	Reverse Primer	Annealing Temperature (°C)	Cycles
12:g.12442525A>G	5'-CATAGCCCA TCCACAGTCCC-3'	5'-TCCAGTAACG TGCTGTGCAA-3'	64	32
12:g.10594033_10594034insAGC AGCTCCAGC	5'-56-FAM- ACACTGT TACCTGGGAAGCG -3'	5'- TCCTTTTTGTCTT CATTTGGTAGAA -3'	57	35
12:g.12303010C>T	5'-TAATGGCAGT GGTAGGCACA-3'	5'-56-FAM- AGGCGAT CTAACCCTCAAT TCC-3'	57	36
12:g.12318425-_12318441insT	5'-TTT CCC AGT CAC GAC GTT GCA GTG GGT CTG CAA GTG TG-3'	5'-M13FAM- GTT TCT TGT AGA CTA GAT TTC TAT AGA ATT GG -3'	57	35
12:g.12318425-_12318441insT	5'- TTT CCC AGT CAC GAC GTT GAT TGT GGT CAC CCC TGG AG-3'	5'-M13FAM- GTT TCT TTT CCC ACT TCT CTG CTT TTG-3'	57	35

Table S3: Variants prioritized for evaluation from filter 1 and their EquCab2 coordinates, polymorphism, Reference SNP ID, gene, variant type, and caller utilized to identify variant.

SNP ID	Reference SNP ID	Position EquCab2	Polymorphism	Gene	Type of variant	Variant caller
12:g.11760227T>G	rs1139122190	12:11760227	T > G	RAPSN	intron variant	Freebayes
12:g.11956103A>G	rs394505241	12:11956103	A > G	AGBL2	intron variant	Freebayes
12:g.12318425-_12318441insT	-	12:12318425-12318441	CTTTTTTTTTTTTTTTA > CTTTTTTTTTTTTTTTTA	PTPRJ	intron variant	Freebayes
12:g.12442525A>G	rs1150980285	12:12442525	A > G	N/A	intergenic region variant	Freebayes and Samtools

Table S4. Variants identified from remapping to EquCab3 and using filter 1 parameters. SNP ID, Reference SNP ID, EquCab3 position, polymorphism, gene, variant type, and caller utilized to identify variant are described.

SNP ID	Reference SNP ID	Position EquCab3	Polymorphism	Gene	Type of variant	Variant caller
12:g.11935134T>C	rs394884702	11935134	T > C	CELF1	intron_variant	freebayes
12:g.12073152A>G*	rs394505241	12073152	A > G	AGBL2	intron_variant	samtools

*SNP identified in both EquCab2 and EquCab3 analysis

Table S5: Variants prioritized for further evaluation using filter two criteria (mapped to EquCab2) and their relative position, alleles, gene, and predicted functional effect.

SNP ID	Reference SNP ID	Position	Polymorphism	Gene Involved	Type of variant	Predicted Effect
12:g.10544757T>C	rs393821033	10544757	T > C	LARGE2	missense variant	Moderate
12:g.12004293C>T	rs395357909	12004293	C > T	FNBP4	missense variant	Moderate
12:g.12064892A>G	rs396307318	12064892	A > G	NUP160	splice region variant, intron variant	Low
12:g.11311198C>G	rs68918770	11311198	C > G	CKAP5	intron variant	Modifier
12:g.11317109A>G	rs68918774	11317109	A >G	CKAP5, CKAP5-LRP4	upstream gene variant, intergenic region	Modifier
12:g.11754791A>C	rs393933135	11754791	A > C	RAPSN, PSMC3-RAPSN	downstream gene variant, intergenic region	Modifier
12:g.11756545T>C	rs395098279	11756545	T > C	RAPSN, PSMC3-RAPSN	downstream gene variant, intergenic region	Modifier
12:g.11757241C>A	rs397265292	11757241	C > A	RAPSN, PSMC3-RAPSN	downstream gene variant, intergenic region	Modifier
12:g.11757280T>C	rs394395559	11757280	T > C	RAPSN, PSMC3-RAPSN	downstream gene variant, intergenic region	Modifier

12:g.11757948C>A	rs397066765	11757948	C > A	RAPSN, PSMC3-RAPSN	downstream gene variant, intergenic region	Modifier
12:g.11760804C>G	rs397233884	11760804	C > G	RAPSN	intron variant	Modifier
12:g.11762360C>T	rs395068088	11762360	C > T	RAPSN	intron variant	Modifier
12:g.11762723G>A	rs395257098	11762723	G > A	RAPSN	intron variant	Modifier
12:g.11763593C>G	rs394453963	11763593	C > G	RAPSN	intron variant	Modifier
12:g.11764018A>T	rs68922985	11764018	A > T	RAPSN	intron variant	Modifier
12:g.11764057C>T	rs397156951	11764057	C > T	RAPSN	intron variant	Modifier
12:g.11764248A>G	rs395668179	11764248	A > G	RAPSN	intron variant	Modifier
12:g.11764903G>C	rs394234717	11764903	G > C	RAPSN	intron variant	Modifier
12:g.11764938A>G	rs394745750	11764938	A > G	RAPSN	intron variant	Modifier

12:g.11765122A>G	rs394078311	11765122	A > G	RAPSN	intron variant	Modifier

Table S6: Prioritized variants from Filter 3, genomic coordinates (EquCab2), alleles, gene, and predicted effect.

SNP ID	Reference SNP ID	Position	Polymorphism	Gene Involved	Type of variant	Predicted Effect
12:g.10448429G>A	rs1140847537	10448429	G > A	SLC35C1	missense variant	Moderate
12:g.10483604G>A	rs782825759	10483604	G > A	CRY2	missense variant	Moderate
12:g.10527154G>A	rs1138376368	10527154	G > A	C11orf94	missense variant	Moderate
12:g.10594033_10594034insAGCAGCTCCAGC	-	10594033	TT > TTGCTGGAGCTGCT	PHF21A	conservative inframe insertion	Moderate
12:g.11185945A>C	rs1143834604	11185945	A > C	ZNF408	missense variant	Moderate
12:g.11187265G>A	rs1145066845	11187265	G > A	ZNF408	missense variant	Moderate
12:g.11189489C>T	rs782896651	11189489	C > T	ZNF408	missense variant	Moderate
12:g.11190608G>T	rs395005478	11190608	G > T	ZNF408	missense variant	Moderate
12:g.11200749A>G	rs68901053	11200749	A > G	F2	missense variant	Moderate

12:g.11219937G>A	rs782886448	11219937	G > A	CKAP5	missense variant	Moderate
12:g.11563067T>C	rs397339518	11563067	T > C	ARFGAP 2	missense variant	Moderate
c.1013C>T*	rs1139682898	11608667	C > T	DDB2	missense variant	Moderate
12:g.11643898G>A	rs68921652	11643898	G > A	MADD	missense variant	Moderate
12:g.11874540A>C	rs1138397738	11874540	A > C	FAM180B	missense variant	Moderate
12:11980715_11980716ins GT	rs1138587553rs 1142374616	11980715	GG > AC	AGBL2	missense variant	Moderate
12:g.11984978T>C	rs396768162	11984978	T > C	AGBL2	missense variant	Moderate
12:g.11990634C>T	rs395199588	11990634	C > T	AGBL2	missense variant	Moderate
12:g.12303010C>T	rs393935064	12004291	C > T	FNBP4	missense variant	Moderate
12:g.12303010G>A	rs1138536580	12303010	G > A	PTPRJ	missense variant	Moderate

12:g.12310371A>G	rs395213493	12310371	A > G	PTPRJ	missense variant	Moderate
12:g.12755911G>T	rs1136731882	12755911	G > T	ENSECAG00000007578	missense variant	Moderate
12:g.12756268G>A	rs1147537747	12756268	G > A	ENSECAG00000007578	missense variant	Moderate
12:g.12756288C>A	rs1147006452	12756288	C > A	ENSECAG00000007578	missense variant	Moderate
12:g.12756349G>T	rs1150502738	12756349	G > T	ENSECAG00000007578	missense variant	Moderate
12:g.12763013T>C	rs1140739982	12763013	T > C	ENSECAG00000007591	missense variant	Moderate
12:g.10565787G>A	rs396254195	10565787	G > A	PHF21A	splice region variant	Low
12:g.10876198C>T	rs396007404	10876198	C > T	CREB3L1	splice region variant	Low
12:g.11170565C>T	rs1143288338	11170565	C > T	ARHGAP1	splice region variant	Low
12:g.11214354T>C	rs1145308378	11214354	T > C	F2	splice region variant	Low

12:g.11239116A>G	rs1147385867	11239116	A > G	CKAP5	splice region variant	Low
12:g.11251848A>G	rs1147814017	11251848	A > G	CKAP5	splice region variant	Low
12:g.11614594T>A	rs395251245	11614594	T > A	ACP2	splice region variant	Low
12:g.11648582_11648588delTCATA	-	11648582	GATCATA > GA	MADD	splice region variant	Low
12:g.11655415T>C	rs1146047742	11655415	T > C	MADD	splice region variant	Low
12:g.11682487T>C	rs1137350264	11682487	T > C	MYBPC3	splice region variant	Low
12:g.12332341C>T	rs68914686	12332341	C > T	PTPRJ	splice region variant	Low

**DDB2* risk variant that was identified previously in Bellone *et al.* 2017

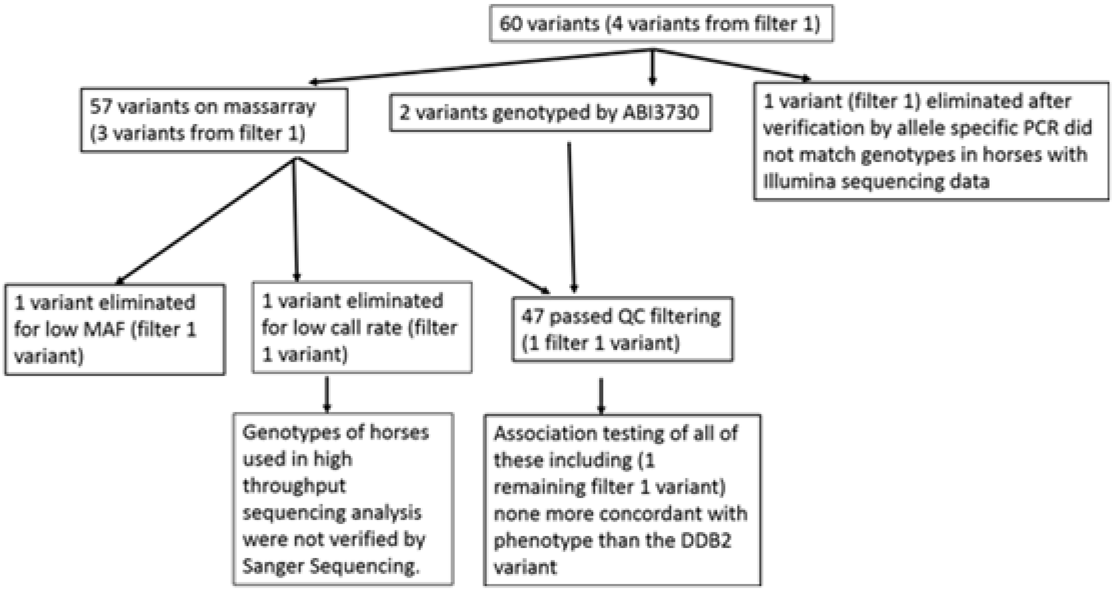

Figure S1: Flow diagram of variants from prioritization through analysis. This chart describes how all of the 60 prioritized variants were genotyped and analyzed. Only 57 were analyzed by MassArray while 3 additional variants were verified or genotyped using allele specific assays.

Table S7: Investigating gender with respect to SCC affection status in Haflingers.

	**Male**	**Female**	**Total**
Ocular SCC Affected	27	32	59
Unaffected	26	35	61
Total	53	67	120
X^2=0.1199, P-value = 0.73			

Table S8: Ocular SCC cases by breed and their *DDB2* (c.1013C>T) genotypic distribution.

Breed	Ocular SCC	***DDB2* Genotype**			Fishers Exact Test P-value
		C/C	***C/T***	***T/T***	
Appaloosa	46	46	0	0	1
Arabian	19	19	0	0	1
Belgian*	25	4	2	19	<0.00001
Haflinger*	59 (12 new samples)	6 (2 new)	8 (2 new)	45 (8 new)	<0.00001
Rocky Mountain Horse*	1	0	0	1	1
Percheron	3	1	2	0	1

*Genotype data reported in *Bellone et al*. 2017, Singer-Berk *et al*. 2018, Knickelbein et al., 2018, and Knickelbein *et al*. *2019*

Table S9: Breeds diagnosed with oral SCC and their respective genotypes for the *DDB2* variant c.1013C>T.

Breed	Oral SCC	***DDB2* Genotype**		
		C/C	***C/T***	***T/T***
Appaloosa	4	4	0	0
Arabian	2	2	0	0
Haflinger	2	1	1	0

.

Table S10: Urogenital SCC sample information and *DDB2* c.1013C>T genotypes.

Breed	Urogenital SCC	***DDB2* Genotype**			Fishers Exact Test P-value
		C/C	***C/T***	***T/T***	
Appaloosa	60	56	4	0	1
Arabian	13	13	0	0	1
